# Who will attack the competitors? How political parties resolve strategic and collective action dilemmas in negative campaigning

**DOI:** 10.1177/1354068815619832

**Published:** 2015-11-29

**Authors:** Martin Dolezal, Laurenz Ennser-Jedenastik, Wolfgang C Müller

**Affiliations:** Department of Government, University of Vienna, Vienna, Austria; Department of Government, University of Vienna, Vienna, Austria; Department of Government, University of Vienna, Vienna, Austria

**Keywords:** Austria, division of labour, negative campaigning, party organization, party roles

## Abstract

Negative campaigning presents parties with a collective action problem. While parties would prefer to have their competitors attacked, potential backlash effects from negative messages mean that individual politicians typically lack the incentives to carry out such attacks. We theorize that parties solve this problem by implementing a division of labour that takes into account the incentives of individual office holders, their availability for campaign activity, and media relevance. Drawing on these arguments we expect that holders of high public office and party leaders are less likely to issue attacks, leaving the bulk of the ‘dirty work’ to be carried out by party floor leaders and general secretaries. Examining almost 8000 press releases issued by over 600 individual politicians during four election campaigns in Austria, we find strong support for our theoretical expectations.

## Introduction

In modern democracies electoral campaigns ought to serve the citizens by allowing the candidates to present themselves, their programs, and their records and to conduct a public debate focused on them (LeDuc et al., 2002). However, parties and candidates take an active role in these debates also by attacking the achievements, plans, and candidates of competing parties. Quite simply, the parties’ strategic objectives are to appear attractive to the electorate and at the same time to reduce the attractiveness of their competitors. The two resulting types of behaviour are called positive and negative campaigning, respectively.

Parties often consider negative campaigning essential to influence the outcome of the election, as the weaknesses of their competitors may otherwise remain unnoticed. Riding effective attacks therefore is a task parties have to accomplish. Yet there is a tension between the two goals of appearing attractive and reducing the attractiveness of others as research has established a backlash effect of negative campaigning (Lau et al., 1999, 2007).^1^ Even though some studies report beneficial effects of attacks (Geer and Lau, 2006), mass media and voters typically dislike them – with the consequence of popularity losses for the attacker. In multiparty systems, attacking politicians and parties may also suffer policy and office costs, as targeted (prospective) coalition partners may be less willing to cooperate. As a consequence, party elites face a disincentive to attack other parties.

In the United States, parties and candidates have resolved this dilemma by farming out attacks, and toxic ones in particular, to outside groups not formally tied to a candidate or party, the so-called (Super) PACs (Brooks and Murov, 2012; Painter, 2014). In European party democracies such farming out of campaign tasks is at best a nascent development as political parties still dominate the contest (Farrell and Schmitt-Beck, 2008). This leaves them with two strategic dilemmas: First, how to attack competitors while keeping the backlash effect for the party at bay? Second, how to overcome the collective action problem that rests in the conflict between collective party gains in terms of discrediting competitors and individual costs in terms of popularity losses with the electorate and poisoned relationships with other parties’ politicians?

To answer these research questions we resort to two rarely connected literatures: that on party organizations and that on political roles. Combining these literatures we begin building what eventually may become a theory of intra-party roles based on various party and public offices in the context of systems with coalition government. From there we derive several unnecessary hypotheses which we test with data from the last four national elections in Austria (2002, 2006, 2008, and 2013), a typical European parliamentary democracy. Parties with diverse ideological backgrounds competed in these elections and the observation period also includes different types of governments: Until 2006, Austria was governed by a centre-right coalition; since then grand coalitions have been in power.

Empirically, we base our study on a content analysis of press releases. We choose this important communication means as it is accessible to a great number of party actors who all should share the collective party goals but face different individual incentives to act upon them. Hence, this source should reveal different degrees of negativity in the campaign communication as a consequence of varying roles.

Our results widely confirm this expectation. Moreover, these differences in the level of negativity are not only observable between subjects but also within subjects, as shown by our analysis of a sub-group of individuals who changed their offices – and thus their expected roles – over time.

## Intra-party roles and campaign communication

Modern democracies are characterized by partisan dealignment and increasing levels of electoral volatility (Dalton and Wattenberg, 2000). Against this background electoral campaigns have greatly gained in importance. It is here where parties present their candidates, ideas for future policies, and records, but also engage with their competitors. This is reflected in a large and growing literature on campaigns and campaigning (Bowler and Farrell, 1992; Brady and Johnston, 2006; Jacobson, 2015; Plasser and Plasser, 2002; Schmitt-Beck and Farrell, 2002; Trent et al., 2011). Much campaigning, this literature has shown, is negative in the sense that its focus is not on the relevant actors’ claimed strengths but their competitors’ alleged weaknesses and faults (Lau and Rovner, 2009; Nai and Walter, 2015).

However, research on negative campaigning has also established a backlash effect: While attacks may hurt the targets, they also harm the attacker (Lau et al., 1999: 856–857; Lau et al., 2007: 1182–1183). Mass media are more likely to report negative messages but journalists may also connect the sender to aspects of politics disliked by the voters. Notwithstanding such a backlash effect, political parties may have no better option than to also campaign negatively. If no one else highlights the weaknesses of their competitors, if, for instance, the mass media display a partisan bias, are docile vis-a-vis incumbents, or simply superficial, there may be no other way to make voters aware about such faults (Geer, 2006). Leaving aside some protest parties, parties as such are unlikely to run entirely negative campaigns. Mixed campaigns with both negative and positive party communication are more likely so that the backlash effect might be contained. In addition to such balancing, we theorize that parties can further minimize the costs of negative campaigning by an intelligent handling of that task.

Parties are collective organizations but organizations can act only through individuals. According to the political entrepreneurial perspective of politics (Laver, 1997), these individuals ‘do not have partisan goals per se’ (Aldrich, 2011: 5). They rather have career and policy goals in government for which the party is an instrument. Individually striving for such goals can lead to results that are inferior to coordinated behaviour and hence not the best collective outcome for political parties. In short, political parties face a collective action problem when it comes to negative campaigning. This leaves us with a double puzzle: How do political parties manage to attack their competitors if individual incentives for such behaviour are lacking? And how do parties as organizations contain the detrimental effects of negative campaigning?

Answering these research questions requires looking into political parties and their campaign communication in some detail. However, the literature on negative campaigning in European party democracies typically uses ‘party’ as the unit of analysis and hence cannot provide an answer to this question (Elmelund-Præstekær, 2008, 2010; Hansen and Pedersen, 2008; Schweitzer, 2010; van Heerde-Hudson, 2011; Walter, 2014; Walter and van der Brug, 2013; Walter and Vliegenthart, 2010; Walter et al., 2014). Nor did researchers who studied (female) party leaders (Walter, 2013) or the behaviour of presidential candidates (Sigelman and Shiraev, 2002) look inside parties. Only a study on communication patterns in a Dutch election campaign provides some intra-party differentiation (de Nooy and Kleinnijenhuis, 2013). Likewise, Schweitzer’s (2010) study of online campaigning compares party leaders to other party representatives.

In contrast, the US literature largely focuses on individual candidates and hence allows for comparing the campaign behaviour of candidates within the same party. Yet, their competitive context is very different. In a way each candidate for legislative office resembles a party that aims for success in the relevant single-member constituency and relies on his or her own campaign organization. Presidential elections, by contrast, rather resemble a team effort as the candidates and their running mates are tied together. In this regard Sigelman and Buell (2003) found some evidence for the ‘conventional wisdom’ that vice-presidential candidates carry the main burden of negative campaigning.

The case of US presidential elections thus suggests some division of labour within a party’s elite in negative campaigning. Such division of labour should be much more systematic in Europe’s strong party organizations. Although the literature on political parties has always displayed a strong interest in issues of organization and intra-party politics it has not dealt with this particular question. While we know much about the internal structures of parties in terms of collective decision-making bodies (Katz and Mair, 1994), the comparative literature on political parties is largely silent on the internal division of labour.

This is even true for the one office given to individuals that has received most attention, that of party leader. Although a sizeable literature exists on party leaders, it is mostly on their election and de-selection rather than what they do in office (Pilet and Cross, 2014). And although their office performance is essential in these processes the literature typically avoids mapping their behaviour but rather draws on external evaluations such as public opinion polls or electoral results. The growing literature on the importance of leaders in elections (Aarts et al., 2011; Bittner, 2011; King, 2002) also rarely focuses on their actual behaviour during campaigns – with the major exception of TV debates – but examines rather stable factors such as their personality or issue positions.

Party statutes may also mention a few more positions given to individuals – such as secretary, financial officer, and keeper of the minutes – but typically they do not describe these jobs in detail. Aldrich (2011: 17–18) provides a basic differentiation based on (a) those who hold elective office (‘office seekers’) and (b) professional communication experts and activists (‘benefit seekers’). Yet the empirical literature has not dealt much with this topic. Regarding the work of party employees, Webb and Kolodny described it as ‘one of the most under-researched fields in the study of political parties’ (2006: 337). We may therefore approach our research question from a different angle.

This perspective is, as Kitschelt (2006: 288, note 281) dubs it, ‘task-directed’ functionalism (which is different from ‘explanatory’ functionalism). In this vein, Schlesinger (1993) takes the competition in elections as the most basic feature in the study of political parties. An ‘electoral imperative’ dictates office-seeking parties a number of tasks. These tasks are different from the more abstract goals of office-seeking, policy-seeking, or vote-seeking (Müller and Strøm, 1999; Strøm, 1990) which Schlesinger reserves for individuals.^2^ He rather provides a list of tasks that need to be fulfilled in the US system, ranging from the declaration of candidacy to behaviour in office (1993: 484–493). One of these tasks is dubbed ‘complex communication’ delineating the need to ‘convince voters’. As indicated above, in modern democracies this often involves discrediting competitors. Discussing different regime types, Schlesinger indicates that the individual incentives of party officials to cooperate in achieving the task of convincing voters differ in unitary (parliamentary) and divided (presidential) systems, with the former ones being more cooperative than the latter. Yet he allows for ‘some independent campaigning’ (1993: 490) of party nominees even in unitary systems if they campaign in geographically delimited areas or compete for different offices. Why would candidates differ under these circumstances? Perhaps because they relate to different reference groups (constituencies) and face expectations closely tied to their respective offices? Such ideas have been especially prominent in the literature on political roles.

Originally, political roles have been given most attention in the study of legislatures (Blomgren and Rozenberg, 2012b; Müller and Saalfeld, 1997; Searing, 1994; Wahlke et al., 1962). Their internal organization builds on a number of formal offices such as president or speaker, committee chair, and party floor leader. These offices are associated with very distinctive formal tasks but they are additionally often related to normative expectations about how the tasks should be performed and how the office holders should behave even beyond their formal duties. Leaving aside the once dominant structural-functional approach (Blomgren and Rozenberg, 2012a: 14–16), contemporary research has integrated the concept of ‘roles’ into the rational choice paradigm. In Searing’s (1994) ‘motivational approach’, roles take a ‘purposive’ nature. They are defined according to the purposes the politicians pursue. Specifically, Searing distinguishes ‘position roles’, tied to specific offices that come with strong expectations about how the role is to be performed, and ‘preference roles’ that are less well defined and allow politicians to pick and choose among potential activities. Strøm (1997, 2012) has continued the move towards a concept of rational behavior. Politicians, he argues, have preferences they try to advance by making strategic decisions about the employment of scarce resources within the given institutional environment and its incentive structures. According to Blomgren and Rozenberg, roles are ‘systematic behaviour’ and ‘actions that repeat over time’ (2012a: 28–29). Roles, then, are rational responses to institutional incentives.

One important aspect in this regard is the degree of partisanship attributed to a specific office. While Wahlke et al.’s famous dichotomy of ‘party man’ vs ‘independent, maverick, nonpartisan’ (1962: 343–376) is not very useful for application in contemporary European party democracies, its underlying dimension is of relevance to our research. Depending on their particular positions in the political system, politicians can be more or less overt partisans in their behaviour.

Modern role theory thus emanates from the parliamentary context. Although this arena remains central in many respects, contemporary politics has moved the political communication battlefield out of it to a large extent. Political actors not only rely on the mass media to transmit their messages, they also approach them directly, tailoring their messages according to the requirements of journalistic transmitters and a mass audience. That is why we build a theory of actor behaviour in this realm.

## Theoretical expectations

Our theorizing starts from formal positions and most basically differentiates them into public vs party offices. These offices can be understood as ‘positional roles’ with regard to our variable of interest, namely negative campaigning. While we develop strong expectations for high offices, the incentives and opportunities for such behaviour are less clear for many lower offices. They rather resemble Searing’s (1994) ‘preference roles’.

In order to predict a politician’s inclination to carry out attacks we need to answer three questions: First, what is their incentive structure to attack competitors? Second, to what extent are they available for genuine party (rather than public office) work? Third, what is their relevance for media, meaning what chance do they have to get their messages reported by the mass media due to their office(s)? Only when these questions are answered in a particular way can we expect the individuals to internalize the party demand upon negative campaigning, to regularly act accordingly, and to achieve effect.

In terms of public offices, parliamentary regimes appear similar enough to allow a straightforward cross-national application, though we expect differences between systems with single-party and coalition governments. We differentiate the following public offices: head of government, cabinet member, and speaker of parliament. In terms of party offices, by contrast, the empirical variation is certainly greater. A cross-national application of our approach would therefore require starting from the conditions we formulate rather than the specific offices we relate to these conditions in the Austrian context. These party offices are: party leader, party floor leader, and party general secretary. With respect to the six public and party offices we additionally consider differences between parties in government and opposition. All other holders of public and/or party office constitute the group of ‘other politicians’.

### Incentive structures

Assuming that politicians are rational actors, the first and most fundamental question concerns the office-related incentive structure for negative campaigning. The distinction between government and opposition is crucial for the definition of some of the public offices and this also impacts on the incentive structure of party offices.

#### Head of government

This office is the main prize of politics in parliamentary systems. For political parties, incumbents (most of the time) are electoral assets that need to be preserved. Clearly, such preservation would also serve the career ambitions of the incumbents. Ascending to statesmanship by meeting with world leaders might help; descending to mere partisan politics by engaging closely with political competitors is more likely to have the opposite effect. At the same time the job of prime ministers is to keep the government running. This means to resolve conflict rather than to forge it in coalition governments. All this suggests that heads of government have strong incentives to avoid negative campaigning.

#### Cabinet member

The incentive structure for cabinet members is similar to that of the head of government. They are among the most visible party representatives and for the sake of the party and their own career they should avoid public opinion backlash. While they are not primarily responsible for the working of the government *tout court*, they clearly contribute to it. Moreover, their own success as ministers may depend on the goodwill of coalition partners. They, therefore, have an incentive not to strain relations with them and to avoid clashing with opposition politicians by riding attacks on them.

#### Speaker of parliament

This office is close to the top of any state’s formal political hierarchy and in most European countries it is met with strong non-partisan role expectations (Jenny and Müller, 1995). While this first and foremost means procedural fairness in the conduct of parliamentary affairs, it is easy to see that credibility for such behaviour may suffer from taking a leading role in partisan attacks. Office holders may also aim for even higher office such as head of state. In constitutional monarchies where this career option is not available, the position of speaker is typically taken by elder statespersons who have grown out of party politics. In any case, speakers of parliament typically have very little motivation to expose themselves to the backlash effect that attacks on opponents produce.

#### Party leader

The party leader is increasingly important as an electoral asset of the party (Aarts et al., 2011; Bittner, 2011; Costa Lobo and Curtice, 2015; McAllister, 2007). He or she has both a party and personal incentive to avoid backlash effects and abstain from negative campaigning. These statements are first and foremost relative to other officials of the same party, allowing some differences between government and opposition parties. Specifically, it is the role expectation of the opposition to criticise and attack the government. We therefore expect that leaders of opposition parties practice less self-constraint in negative campaigning. Their taking a more active part in attacks may also be a necessity if journalists tend not to report what less prominent opposition politicians say.

#### Party floor leader

Although the basis of this office is a public one – being a member of parliament – leading the parliamentary party is a genuine party office. Leading the party in parliamentary battles without doubt requires attacking competitors. Yet being the party’s spearhead is not the only task associated with this office, in particular in government parties where floor leaders are part and parcel of the machinery of government. In coalitions this task typically includes the parliamentary coordination with the other government parties. While floor leaders of opposition parties have strong incentives to attack all their opponents, those of government parties might be interested in smoothing rather than straining intra-coalition relations and to concentrate their fire on the opposition.

#### Party general secretary

In his characterization of party secretaries even in democratic parties, Duverger refers to Lenin’s *What is to be done?* There, Lenin praised the secretaries’ ‘total and permanent devotion to the party’; together with their availability (see below) this makes them the party’s ‘real agitators’ (see Duverger, 1959: 155). Lenin’s revolutionary avant-garde clearly represents the extreme end but, according to Duverger, more than a kernel of truth also for party secretaries in democratic parties. In addition to the material rewards they receive from the party there are also symbolic rewards from the party activists who are believed to be more radical than passive party members and voters and often appreciate offensive behaviour of their leaders (see May, 1973). Despite large variation in their internal organization, most European parties feature a functional equivalent of the party secretary, usually called general secretary or secretary general (e.g. in Germany, Austria, Denmark, Sweden, Finland, Spain, Ireland, or the UK), party secretary (Belgium), or party president (the Netherlands). The job description usually features the day-to-day operation of the extra-parliamentary party organization, in many cases including the management of election campaigns and speaking on behalf of the party.

#### Other politicians

The offices we have singled out should comprise a large share of politicians who contribute to public campaign discourse. The remaining politicians include MPs, parliamentary candidates, and sub-national office holders. They tend to have less relevance for media and quite heterogeneous incentive structures to participate in the campaign and attack competitors in particular.

While these expectations seem plausible for the public and party offices per se, real world politics is somewhat more complex as several individuals combine party and public offices. In such circumstances we expect the incentives from public office to be stronger. This is in line with Aldrich who argues that politicians take the party as ‘the instrument for achieving’ their ‘more personal and fundamental goals’ (2011: 5) in public offices.

### Availability for party activity

The second important question is to what extent office holders are available for party activity. While making a contribution to the public political debate may not require much time per se, the precise timing of such interventions is often crucial. Reacting too late may mean that the public floor de facto has been left to the competitors. A too late response may miss the editorial deadlines of important mass media and fail to balance or override messages from political competitors. Availability therefore to a large extent means time flexibility and accessibility for the party’s campaign strategists and ‘war room’ managers.

Such availability is severely limited in the case of members of the executive who may be bound up in meetings or international travel (especially to Brussels), duties that do not vanish in campaign periods. Holders of high parliamentary office – the presidents of parliament and the floor leaders – should display much greater availability, as the parliament typically is not in session when the election approaches. This is probably less true for MPs, many of whom will have to combine private occupation and constituency campaigning.

With respect to party office holders, the party secretaries again are most likely available. Contrary to other politicians they are almost permanently present in the capital and the party headquarters. Again Duverger’s reference to Lenin’s work is telling: Being employed by the party, they can serve it ‘with no interruption or hindrance due to external cares’ (Duverger, 1959: 155).

### Relevance for media

The classic criteria of ‘newsworthiness’ applied by journalists include the prominence of the sender in addition to the newness and negativity of the message (O’Neill and Harcup, 2009). The most likely source of prominence is high public office followed by high party office.

Three groups of actors seem plausible: The top group includes the head of government and the (other) party leaders and top candidates respectively. A middle group comprises the members of the cabinet, the speakers of parliament, the parliamentary floor leaders, the party secretaries, and leading sub-national executive officers. A third group, finally, consists of MPs, other sub-national office holders, and candidates without public office.

We can now bring the discussions of the three questions together. Clearly, the incentives to attack constitute the most important factor. Here we see that the holders of high public office have no incentive to attack competitors. Even party leaders have little incentive to do so, though leaders of opposition parties and those who are serious contenders for the office of prime minister should be more prone to attack. Parliamentary floor leaders, especially those of opposition parties, and the parties’ general secretaries in particular are the offices that we see most predetermined to ride attacks against competing parties. Conveniently, these offices, and the general secretaries in particular, are also endowed with the required time resources and relevance for media to lend effectiveness to such behaviour. Table 1 summarizes these expectations.

**Table 1.** Political offices and the propensity for negative campaigning.

Office Type	Political office	Government	Opposition
Public	Head of government	Low	—
	Cabinet member	Low	—
	Speaker of parliament	Low	Low
Party	Party leader	Low	Medium
	Party floor leader	Medium	High
	Party general secretary	High	High

## Data and methods

The present article is based on a content analysis of party press releases. This source, to the best of our knowledge, has been hardly used in the study of negative campaigning^3^ even though it has two general advantages: First, it is under the direct control of the sender and thus adequately represents a party’s campaign strategy. Studies based on media reports, by contrast, might suffer from the media’s negativity bias giving conflict a higher chance to get reported (Elmelund-Praestekaer and Molgaard-Svensson, 2014; Hansen and Pedersen, 2008; Ridout and Walter, 2015). Second, press releases are issued frequently and continuously during a campaign and therefore capture its dynamics (Dolezal et al., 2015). For the present article this source is best suited because of a further characteristic: In contrast to TV debates or TV spots, press releases are not an exclusive means for the parties’ top candidates. Press releases allow for studying the campaign communication of a much broader range of party representatives. Naturally, leading politicians can easily use other means of communication such as interviews in newspapers or TV news shows. However, press releases typically follow these channels and distribute the messages provided to a broader media audience.

In Austria, press releases are distributed via the APA, the national news agency. They are called ‘OTS-Meldungen’ (Original Text Service-Messages) and are freely available through a website (www.ots.at). This centralized distribution increases the messages’ importance especially for journalists who are their main audience. Research has demonstrated that press releases strongly influence news coverage in many countries, including Austria (Haselmayer et al., 2015; Seethaler and Melischek, 2014).

For each of the four campaigns, we selected all press releases sent during the last six weeks of the campaign by the parties represented in parliament before and/or after the election. We not only included press releases sent by the parties’ central offices but also by their parliamentary groups or regional branches. In a further step we manually de-selected all press releases that only informed about coming events (e.g. press conferences or campaign rallies) or provided technical information (such as links to pictures of candidates or audio content). Note that we deliberately do not include press releases distributed by ministries. These releases might have a partisan ‘touch’ but they are rarely negative. In 2013 we only found one cabinet member using this channel to attack an opponent.

All in all we collected 7858 press releases from seven parties. Apart from the SPÖ (Social Democratic Party of Austria) and the Christian-democratic ÖVP (Austrian People’s Party) these parties include two populist radical right parties, the FPÖ (Freedom Party of Austria) and its split-off, the BZÖ (Alliance for the Future of Austria), the Greens, the liberal NEOS (NEOS–The New Austria), and the populist Team Stronach. While the SPÖ, ÖVP, FPÖ, and Greens were present in all four campaigns, the BZÖ was founded in 2005. The NEOS as well as Team Stronach, by contrast, are new parties and only competed in the 2013 election (Dolezal and Zeglovits, 2014; Kritzinger et al., 2014).

In the content analysis we apply a relational method that captures the relationship of actors (‘subjects’) with issues or other actors (‘objects’). A variable called ‘predicate’ connects them and records their relation as either positive (1), negative (–1), or neutral (0) (see Appendix for examples). This method goes back to the work of Kleinnijenhuis and his collaborators (e.g. Kleinnijenhuis and Pennings, 2001) and was also used in comparative research on election campaigns and public debates (Kriesi et al., 2008, 2012). The Austrian National Election Study (AUTNES) has developed this approach further and uses it for various types of political texts, e.g. party manifestos (Dolezal et al., 2014, 2016).

Given the high number of press releases we only coded their title. However, because of the length of the headings (a maximum of 138 characters set by the OTS-system) and the high quality with which most press releases are written, the content of the titles perfectly captures the basic message of most press releases. What is more, press release titles are the main selection criterion for journalists (only titles and subtitles are visible when journalists scroll through the APA system), thus our measure registers whether party actors choose to make the attack the main point in their communication.^4^

For the present article we define any negative relation between subject and object actors, thus any form of criticism, as negative campaigning (e.g. Geer, 2006: 26). Every press release is therefore coded as 1 ‘attack’ or 0 ‘no attack’. For both the subjects (i.e. the senders) and the objects (the targets) names and organizational affiliation (typically to a political party) were coded so that we can easily identify the individual politicians who held public or party offices. Of course, in an archetypical party democracy such as Austria it is natural to find some overlap between party and public offices. Parties reserve the highest public office available to them for their leaders. Therefore, leaders of government parties typically take positions in cabinet (mostly as Chancellor or Vice-Chancellor), whereas opposition party leaders usually assume the position of party floor leader in parliament.

Apart from the office variables, we also control for gender (as men and women are sometimes expected to differ in terms of negative campaigning), government status, and the week of the campaign (as campaigns may systematically vary in emphasis on individuals and attacks over their course).

## Analysis

Media relevance, as argued above, is the precondition for any communication strategy based on press releases; otherwise journalists would simply neglect them. Results from a content analysis of the news coverage of the 2013 campaign (AUTNES MedienManuell, 2013; Schönbach et al., 2014) demonstrate the high media presence of the politicians holding the six offices we are especially interested in. Even though in 2013 these members of the political elite comprised only 33 individuals (or four percent of all individuals recorded in the content analysis), they were mentioned in no less than 54 percent of all articles or television pieces analysed. In 2008 individuals belonging to this group were coded as ‘main actors’ in 40.7 percent of the articles (AUTNES MedienManuell, 2008).

**Figure 1.**
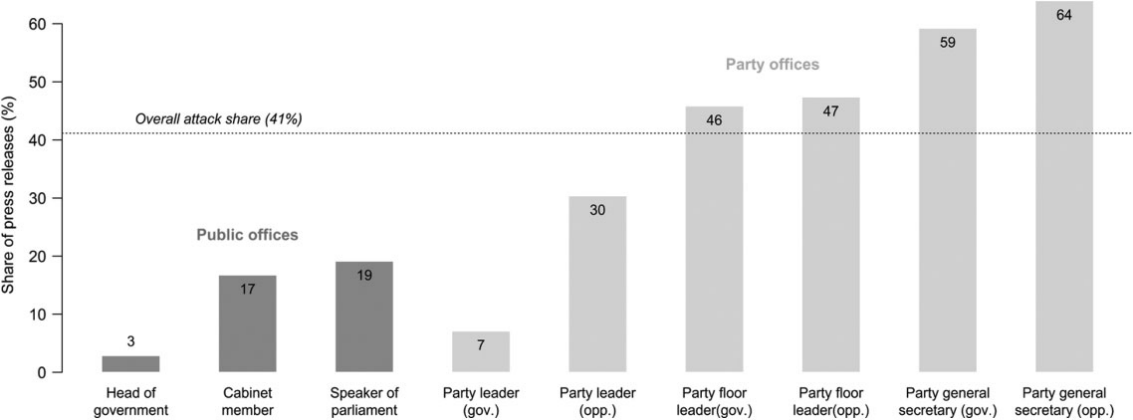
Attack shares by political office. Note: A more detailed breakdown of the number of observations and attack shares by office and election year is presented in the Appendix (Table A1).

Figure 1 presents the level of negativity by political office. Heads of government and leaders of parties in government (i.e. Vice-Chancellors) almost completely refrain from attacking opponents. Other holders of high public office in government and parliament exercise similar levels of restraint. Opposition party leaders are somewhat more likely to direct negative messages at their opponents, yet still stay below the average level of negativity. Party floor leaders are just above average, yet clearly not as aggressive in their messaging as party general secretaries.

To see whether these results hold in a multivariate test, we present a binary logistic regression with random effects at the party-election level (Table 2) to account for structural factors that remain constant for each party during a campaign. The reference category for the political office predictors is the set of non-elite politicians that make up the majority of all senders in the press release data.

**Table 2.** Regression analysis.

Variable	Coefficient	S.E.	Odds ratio
Head of government	–1.839***	0.514	0.159
Speaker of parliament	–0.998***	0.220	0.369
Cabinet member	–0.902***	0.131	0.406
Party leader (gov.)	–1.358***	0.239	0.257
Party leader (opp.)	–0.886***	0.125	0.412
Party floor leader (gov.)	0.0730	0.192	1.076
Party floor leader (opp.)	0.703***	0.135	2.019
Party general secretary (gov.)	0.785***	0.105	2.192
Party general secretary (opp.)	0.984***	0.0968	2.675
Female sender	–0.247***	0.0569	0.781
Party in government	0.0170	0.124	1.017
Week-of-campaign dummies	Yes		
Constant	–0.218*	(0.100)	0.804
N	7858		
Intraclass correlation	0.0161		
σ_u_	0.232		

*Note:* Figures are raw coefficients and corresponding odds ratios from a binary logistic regression with random effects at the party-election level; *p < 0.05, **p < 0.01, ***p < 0.001.

All groups except the government party floor leaders display statistically significant differences from the reference category, with public offices and party leaders displaying negative coefficients and the remaining party offices exhibiting positive effects. The odds ratios suggest large differences between the groups, with heads of government and government party leaders showing the lowest levels of negativity, and opposition floor leaders and party secretaries the highest propensity of attacking.

**Figure 2.**
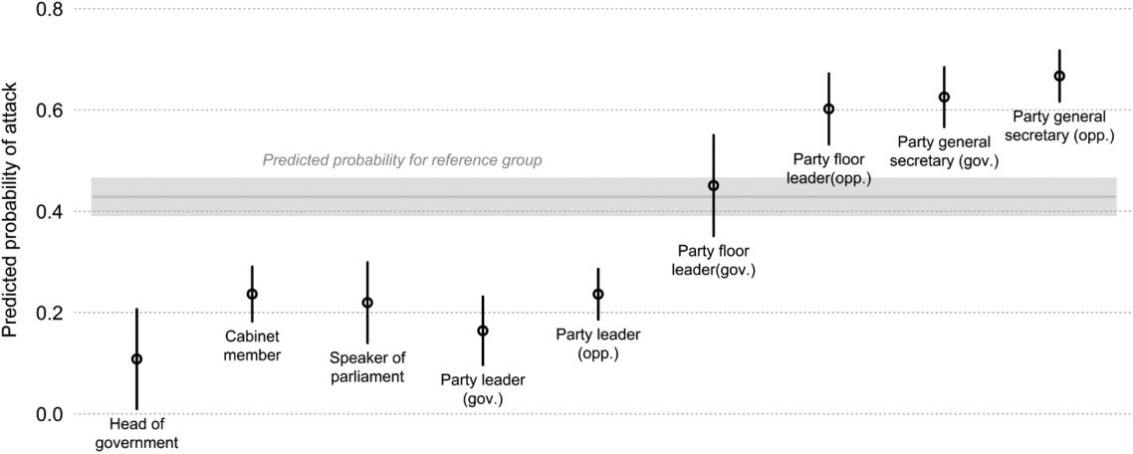
Predicted probabilities of attacking by office. *Note:* Predicted probabilities calculated based on regression in Table 2; all other variables held constant at mean or mode; the government dummy was set to one for categories that coincide with government status; 95 percent confidence intervals shown.

To make effect sizes comparable, we present predicted probabilities from the regression model (Figure 2). Four groups emerge: Heads of government are clearly least likely to attack. A somewhat higher probability of attacking is displayed by party leaders, cabinet members, and speakers of parliament. Next, government party floor leaders exhibit a level of negativity that is indistinguishable from that of the reference group. Opposition party floor leaders and party secretaries have the highest probabilities of attacking.

Taken together, these results largely confirm our expectations. Politicians in high public offices that come with expectations of non-partisanship are least likely to attack, whereas somewhat lower-ranking positions that are also more partisan in nature induce higher levels of negativity. Also, government participation dampens negativity for all party offices (although the differences are not statistically significant for general secretaries). These marked differences according to role expectations are especially relevant as we only included press releases distributed by partisan channels – discarding all official government channels such as ministries which would increase the differences even more.

One criticism that could be levelled against our approach is that the willingness to engage in attack behaviour varies primarily across individuals, and this variation may lead to self-selection (or selection by others) into positions that come with specific role expectations. In order to demonstrate that our findings are robust to these concerns, we take advantage of the fact that many individuals moved into, out of, or between high offices in our observation period. We can thus additionally test our expectations on a smaller sample of observations where the same individuals perform different roles. To arrive at this subgroup we identify all subjects that assume more than one role (including the reference category) across the four election campaigns. In total, the pool of office switchers comprises 41 individuals (see Table A2 in the Appendix) producing over 2300 press releases.

Table 3 presents the same regression model as in Table 2, but with fixed effects at the level of individuals. Thus, all variation left to explain is within individuals switching between offices (we therefore drop the gender variable which is fully accounted for by the fixed effects).^5^

**Table 3.** Regression analysis – office switchers only.

Variable	Coefficient	S.E.	Odds ratio
Head of government	–1.687**	0.586	0.185
Speaker of parliament	–0.837**	0.309	0.433
Cabinet member	–1.011***	0.287	0.364
Party leader (gov.)	–1.524***	0.347	0.218
Party leader (opp.)	–1.541***	0.390	0.214
Party floor leader (gov.)	0.200	0.425	1.221
Party floor leader (opp.)	0.203	0.257	1.225
Party general secretary (gov.)	–0.00163	0.428	0.998
Party general secretary (opp.)	1.196**	0.456	3.305
Week-of-campaign dummies	Yes		
Individual-level fixed effects	Yes		
Constant	0.475*	0.222	1.608
N	2335		
McFadden’s R^2^	0.178		
Log likelihood	–1304.7		

*Note:* Figures are raw coefficients and corresponding odds ratios from a binary logistic regression with fixed effects at the individual level; two individuals drop from the analysis due to all negative outcomes; press releases with two individuals as subjects discarded; *p < 0.05, **p < 0.01, ***p < 0.001.

As Table 3 shows, the results are very similar to our analysis of the full sample. Compared to the reference category, holders of public office and party leaders use negative messages to a much lesser extent. The coefficients and odds ratios for the party floor leaders imply little difference compared with the reference group. The same conclusion can be drawn for general secretaries in government parties. By contrast, opposition party general secretaries are significantly more negative than the comparison group and thus constitute the group most prone to attack in our subsample of office switchers. These results strengthen our conjecture that the attack patterns observed in the data are not driven by self-selection of more or less aggressive types of individuals into different political roles, but by a strategic division of labour within parties.

## Conclusion

This article builds on and contributes to the literatures on political roles and party organizations in election campaigns. Our core argument holds that parties have good reasons to implement a division of labour regarding negative campaigning. While most parties clearly prefer to have their competitors attacked during election campaigns, the incentives for individual politicians to carry out such attacks are limited. As our analysis shows, parties respond to this collective action problem by shifting the bulk of the ‘dirty work’ away from party leaders and public office holders towards the holders of genuine party positions that come with more partisan role expectations.

In the context of parliamentary systems with coalition governments, the collective interest of the party is served by delegating attacks to the offices of party floor leader and, in particular, general secretary. The latter are part of the party leadership (most often) by means of appointment and therefore remain accountable to the party leader. At the same time, the party compensates them financially and controls their further political career. More than half of all general secretaries in our sample were promoted to ministerial positions after their party entered government. They thus have a personal incentive to attack, if this is part of the party’s strategy. Delegating much of the attacks to them allows other party elites to largely stay free from such behaviour. They thereby follow their personal motivations and, at the same time, do what is in the collective interest of the party.

It is worth pointing out that the effect sizes reported in the regression models are substantial – especially when considering that the large sample size of almost 8000 reduces the chance that random noise produces such huge differences. Moreover, the analysis of a subset of party elites that switch offices between elections strengthens the claim that the observed differences are, in fact, caused by the intra-party division of labour and are not due to self-selection.

Our study is a first step in building a theory of party offices and is limited to party campaign behaviour. While campaigning is a vital party activity, further analyses should expand the scope of analysis to other realms. Policy innovation may allow for a rather straightforward extension of our theoretical reasoning. When parties want to change course on an issue, for instance to expand their electoral appeal, approach potential coalition partners, or because they now consider earlier ideas unworkable, they may face a problem similar to that inherent in riding attacks. Departing from long-standing and firmly held positions can undermine a party’s public image and electoral credibility with traditional voter groups and cause uproar internally. In such uncertainty, a division of labour might be testing the viability of the new policy first by one high-ranking official, for instance a minister or party policy specialist, airing it before the party leader throws his or her authority behind it. Similar to negative campaigning, policy innovation constitutes a collective action problem. While beneficial to the party if successful, it also involves risks. A division of labour similar to the one analysed in this article can resolve this dilemma.

As is true for all single-country studies, there are, of course, limits in how far we can generalize from our findings. However, since Austria is fairly typical of most West European parliamentary democracies regarding party system and party organizational characteristics, we are confident that a similar division of labour is present in many parties in other countries. Even if individual incentives and role expectations may vary somewhat between countries and parties, there are strong reasons to assume that campaign communication will be strongly diversified between holders of different public and party offices.

## Appendix

**Table A1.** Number of press releases and attack shares by office and year.

	2002		2006		2008		2013	
	N	% attacks	N	% attacks	N	% attacks	N	% attacks
Head of government	96	3.1%	33	0.0%	2	0.0%	43	4.7%
Speaker of parliament	38	23.7%	59	13.6%	43	23.3%	1	0.0%
Cabinet member	60	18.3%	62	21.0%	263	15.6%	140	16.4%
Party leader (gov.)	104	3.8%	100	14.0%	116	4.3%	74	6.8%
Party leader (opp.)	142	20.4%	138	34.1%	84	33.3%	110	36.4%
Party floor leader (gov.)	25	52.0%	18	50.0%	42	52.4%	35	31.4%
Party floor leader (opp.)	62	56.5%	62	61.3%	85	42.4%	135	40.0%
Party general secretary (gov.)	172	56.4%	103	65.0%	104	50.0%	114	66.7%
Party general secretary (opp.)	143	76.9%	188	67.6%	218	57.8%	56	42.9%
Other	1157	41.8%	1273	42.6%	1506	41.6%	1211	38.5%

*Note:* Sum of N per election is somewhat greater than total number of press releases because a minority of press releases have two subjects.

Table A1. The table presents the number of press releases issued by each group of office holders in each campaign. Percentages refer to the share of releases that contained an attack. The low N for head of government in 2008 is due to the fact that the incumbent Chancellor, Alfred Gusenbauer (SPÖ), was ousted as party leader and top candidate weeks before the election. The low N for speaker of parliament in 2013 is due to the fact that one of the three individuals (Barbara Prammer, SPÖ) was terminally ill, and the other two (Fritz Neugebauer, ÖVP, and Martin Graf, FPÖ) had fallen out of grace with their parties and not been re-nominated as parliamentary candidates.

**Table A2.** Office switchers by office and year.

Name	2002	2006	2008	2013
Bartenstein, Martin	minister	minister	minister	other
Berger, Maria	other	other	minister	
Bucher, Josef		other	other	party leader & party floor leader
Bures, Doris	general secretary	general secretary	general secretary	minister
Darabos, Norbert		general secretary	minister	general secretary
Faymann, Werner	other	other	minister & party leader	head of government & party leader
Fekter, Maria	other	other	minister	minister
Glawischnig, Eva	other	other	speaker of parliament	party leader & party floor leader
Gusenbauer, Alfred	party leader & party floor leader	party leader & party floor leader	head of government	
Hahn, Johannes	other	other	minister	
Haubner, Ursula		minister	other	other
Haupt, Herbert	party leader & minister	other		
Heinisch-Hosek, Gabriele	other	other	other	minister
Karl, Beatrix			other	minister
Khol, Andreas	party floor leader	speaker of parliament	other	other
Kopf, Karlheinz	other	other	other	party floor leader
Kranzl, Christa	other	other	minister	
Kukacka, Helmut	other	minister	other	
Kuntzl, Andrea	general secretary	other	other	other
Lopatka, Reinhold	other	general secretary	minister	minister
Marek, Christine	other	other	minister	other
Matznetter, Christoph		other	minister	other
Mikl-Leitner, Johanna	other		other	minister
Missethon, Hannes		other	general secretary	
Mitterlehner, Reinhold			other	minister
Molterer, Wilhelm	minister	party floor leader	minister & party leader	
Morak, Franz	minister	minister	other	
Neugebauer, Fritz			other	speaker of parliament
Petzner, Stefan		other	general secretary	other
Platter, Günther		minister	other	other
Prammer, Barbara		other	speaker of parliament	speaker of parliament
Prokop, Liese	other	minister		
Rauch-Kallat, Maria	general secretary	minister	other	
Scheuch, Uwe		general secretary	other	
Schieder, Andreas		other	minister	minister
Schüssel, Wolfgang	head of government & party leader	head of government & party leader	party floor leader	
Silhavy, Heidrun	other	other	minister	
Spindelegger, Michael	other	other	speaker of parliament	party leader & minister
Strache, Heinz-Christian	other	party leader	party leader & party floor leader	party leader & party floor leader
Strutz, Martin	other	other	general secretary	
Westenthaler, Peter		party leader	party floor leader	

Table A2. lists the 41 office switchers and the positions they held across the four election campaigns. Junior ministers are coded as ministers.

### Coding procedure

In the following, we provide some examples of press releases to explain our coding procedure in more detail. We always present the original title, an English translation, the ID of the press release, and the values we record for actors and their relations. Note that press release titles often use informal language and shorthand expressions.

In our relational content analysis we differentiate the subject (the actor producing the message), the object (the actor being addressed, called ‘object actor’), and the predicate (a numerical variable capturing the kind of relation between subject and object). We also record the substantive issue of the press releases as well as additional variables such as references to track record and justification claims of issue positions. However, as these aspects are not relevant for the present article we only explain how we capture relations between political actors.

When coding press releases we record up to two subjects and three object actors. In around six percent of all press releases we find two individuals as subjects. For each actor we record his or her name and organizational affiliation (if it is an individual actor), for collective actors we record the name of the organization. In most cases the subject of a press release is an individual whereas the objects comprise individual as well as collective actors. The title typically does not include the first name of actors. Coders typically find this information in the first paragraph of the press release.

#### Example 1

In the first example an individual actor attacks two collective actors, i.e. parties.

- BZÖ-Petzner: FPÖ und SPÖ stecken in Kärnten tief im Korruptionssumpf fest
- BZÖ-Petzner: FPÖ and SPÖ are stuck in a swamp of corruption in Carinthia
- ID: OTS_20130819_OTS0126

**Table:**

Subject actor(s)		Relation (‘predicate’)	Object actor(s)	
Organization	Name		Organization	Name
BZÖ	Petzner, Stefan	–1	FPÖ	—
		–1	SPÖ	—

#### Example 2

During election campaigns, relations between actors from different parties are mostly negative. Positive relations primarily exist between actors from the same party. In this example a candidate of the ÖVP praises his own party.

- Steindl: ÖVP hat die Konzepte für mehr Arbeitsplätze
- Steindl: ÖVP has the concepts for more employment
- ID: OTS_20130819_OTS0133

**Table:**

Subject actor(s)		Relation (‘predicate’)	Object actor(s)	
Organization	Name		Organization	Name
ÖVP	Steindl, Franz	+1	ÖVP	—

#### Example 3

In the following example an individual (male) politician attacks an individual (female) politician. The reference to two colours refers to a potential coalition of the Christian democratic ÖVP (‘the blacks’) and the populist radical right FPÖ (‘the blues’).

- Schieder zu Fekter: ‘Schwarz-Blau’ ist der Finanzministerin wichtiger als Österreich
- Schieder to Fekter: For the finance minister ‘Black-Blue’ is more important than Austria
- ID: OTS_20130822_OTS0183

**Table:**

Subject actor(s)		Relation (‘predicate’)	Object actor(s)	
Organization	Name		Organization	Name
SPÖ	Schieder, Andreas	–1	ÖVP	Fekter, Maria

#### Example 4

In this example an individual actor who belongs to ‘Team Stronach’ attacks the environment minister whose party affiliation is not mentioned in the press release. In such a case coders have to ‘google’ or use their expertise.

- Fukushima – Stronach/Tadler: Berlakovich beweist seine Ahnungslosigkeit
- Fukushima – Stronach/Tadler: Berlakovich demonstrates his ignorance
- ID: OTS_20130904_OTS0220

**Table:**

Subject actor(s)		Relation (‘predicate’)	Object actor(s)	
Organization	Name		Organization	Name
Team Stronach	Tadler, Erich	–1	ÖVP	Berlakovich, Nikolaus

#### Example 5

Here, Maria Fekter, the ÖVP’s finance minister, attacks the SPÖ, her party’s coalition partner.

- Fekter: SPÖ gefährdet akut Mittelstand und Wohlstand
- Fekter: SPÖ endangers small and medium-sized business and prosperity
- ID: OTS_20130907_OTS0045

**Table:**

Subject actor(s)		Relation (‘predicate’)	Object actor(s)	
Organization	Name		Organization	Name
ÖVP	Fekter, Maria	–1	SPÖ	—

## Notes

1. To the best of our knowledge, no studies on the backlash effect have been conducted for European multiparty systems (Lau et al., 2007; Nai and Walter, 2015: 26).

2. We will argue that individual ambitions may or may not be aligned with performing the tasks.

3. The only exceptions we are aware of are Schweitzer’s (2010) study of campaigning in Germany and Benoit’s ‘functional analysis’ of campaign communication (e.g. Cho and Benoit, 2006).

4. We have coded negative references in subtitles for a subset of observations (for the main government and opposition parties in 2013, SPÖ and FPÖ). In both cases, the share of attacks increases by 10 percentage points. Identifying attacks based on the full text corpus would further increase this proportion, possibly to a point where almost all press releases would have to be counted as attacks.

5. Importantly, we are not concerned with the sequence of offices that individuals assume. We are only interested in the differences in attack behavior that emerge between offices.
